# Antioxidative activity and protein profile of skim milk of *Gaddi* goats and hill cattle of North West Himalayan region

**DOI:** 10.14202/vetworld.2019.1535-1539

**Published:** 2019-10-05

**Authors:** Vinesh Sharma, Birbal Singh, Rinku Sharma, Jyoti B. Dhar, Neelam Sharma, Gorakh Mal

**Affiliations:** 1 Biochemistry Laboratory, Indian Council of Agricultural Research-Indian Veterinary Research Institute, Palampur, Himachal Pradesh, India; 2 Department of Chemistry and Biochemistry, Chaudhary Sarwan Kumar Himachal Pradesh Krishi Vishvavidyalaya, Palampur, Himachal Pradesh, India

**Keywords:** antioxidants, *Gaddi* goats, hill cattle, milk proteins, skim milk, sulfate-polyacrylamide gel electrophoresis

## Abstract

**Aim:**

This study was aimed at evaluation of antioxidative activity, protein profile, and vitamins content of milk of *Gaddi* goats, local non-*Gaddi* goats, hill cattle, and Jersey crossbred cattle.

**Materials and Methods:**

Total phenol, antioxidant activity measured as 2, 2-diphenyl- 1-picrylhydrazyl radical scavenging capacity, total protein, and vitamins were estimated in milk samples by spectrophotometric methods. Milk protein profiles were studied by sulfate-polyacrylamide gel electrophoresis.

**Results:**

Total phenol, antioxidant activity, and total protein were higher in indigenous hill cattle skim milk. Average protein content in raw skimmed milk was 1.33±0.01, 1.03±0.02, 0.76±0.05, and 0.81±0.01%, in indigenous hill cattle, Jersey crossbred cattle, non *-Gaddi* goat, and *Gaddi* goat, respectively. Three proteins of 19.01, 22.08, and 32.96 kDa were observed in *Gaddi* goat, but not in non *-Gaddi* goat skim milk. Furthermore, the above proteins were absent in cattle skim milk. Two proteins of 15.56 and 25.06 kDa were found in local hill and crossbred cattle skimmed milk, but were absent in goat skimmed milk. Vitamin C content was the lowest in *Gaddi* goat milk and the highest in Jersey crossbred cattle milk.

**Conclusion:**

It is envisaged that bioactive metabolites in the milk of *Gaddi* goats and hill cattle might offer anti-aging and beneficial health effects.

## Introduction

Milk is an important source of complex ­proteins, enzymes, and peptides of diverse biological activities [1]. Milk proteins assist in micelle formation, uptake of some nutrients, including trace elements, and vitamins and are a class of biomolecules with protective and immunomodulatory activity [2]. Skim milk contains proteins, carbohydrates, vitamins, and minerals, but lacks lipid and fat-soluble components. Milk proteins serve as important supplementary nutraceuticals with an important role in decreasing cardiovascular disease, cancer, and metabolic diseases [3]. Casein-derived peptides and amino acids are regarded as dietary supplements and used in pharmaceutical formulations [4]. Whey is a promising source of branched-chain amino acids which are used to fuel working muscles and stimulates protein synthesis.

Indian native goats and cattle are invaluable genetic resources [5]. Among native goats, *Gaddi* goat, also known as “White Himalayan goat” is predominantly a migratory and economically important multipurpose goat breed of high altitude in North West Himalayan Region. They are reared primarily for meat, fiber, and milk by the native “*Gaddi*” shepherds [1,6,7]. Hill cattle are dwarf to medium size, economically important, and less explored livestock species for quality of milk. The animals are adapted to high hill- geography and native forages. There are many comparative studies on milk composition among different animal breeds. However, our study gives a comparative profile of biomolecules of nutraceutical importance of cattle and goat milk.

This study was aimed at evaluation of antioxidative activity, protein profile, and vitamins content of milk of *Gaddi* goats, local non-*Gaddi* goats, hill cattle, and Jersey crossbred cattle.

## Materials and Methods

### Ethical approval

The current study was carried out as a part of Institute Research Project duly approved by the Research Committee.

### Chemicals, culture media, and plastic ware

All chemicals, reagents, and supplements were of analytical grade purchased from CDH biochemical, India. Protein molecular markers and 2, 2-Diphenyl- 1-picrylhydrazyl (DPPH) were from Sigma (St. Louis, Missouri, USA). Plasticware was purchased from HiMedia Lab Mumbai (India). Membrane filters (0.45 µm) and silica gel coated aluminum plates (thin-layer chromatography [TLC] silica gel 60, size: 20 cm × 20 cm) were purchased from Merck KGaA (Germany).

### Milk samples and their processing

Lactating goats (at lactation stage of 30-40 days) were maintained under extensive livestock rearing system while hill and Jersey crossbred cows were maintained under a semi-intensive management system.

Milk from *Gaddi* goats, local non *-Gaddi* goats, hill cattle, and Jersey crossbred cows was collected in sterile plastic containers. The samples were held on ice, transported to the laboratory, and were processed to separate skim milk. Raw milk was centrifuged at 12,000 rpm for 45 min at 4°C. Fat micelles were discarded, and skimmed milk was obtained.

### Antioxidative profiling

Potential antioxidant milk components were extracted according to Alyaqoubi *et al*. [8]. Total phenol was estimated as per Alyaqoubi *et al*. [8]. DPPH free radical scavenging activity of skimmed milk was estimated as per protocols described by Brand-Williams *et al*. [9] with slight modifications. Analysis of qualitative DPPH antioxidant activity was done as per Mal *et al*. [1]

### Protein profiling

Protein concentration in skim milk was estimated, according to Lowry *et al*. [10]. Sodium dodecyl sulfate-polyacrylamide gel electrophoresis (SDS-PAGE) was performed according to the standard method [11] using 12.5% separating and 5% stacking gel. The gels were stained with coomassie brilliant blue R-250 and destained using 12% (*v/v*) methanol and 7% (*v/v*) acetic acid solution. Gel images were recorded using AlphaImager Gel Doc System and analyzed by AlphaView SA Software (ProteinSimple). Size of the proteins in the milk samples was determined by comparing the electrophoresed proteins with protein molecular markers (Sigma, Cat. no. S8445, ranged from 6.5-205 kDa).

### Vitamin profiling

Vitamins A, C, and E were determined by standard protocols. The method of Rutkowski *et al*. [12] was used to estimate Vitamin A, Roe and Kuether [13] was used to estimate Vitamin C, and Rutkowski *et al*. [14] was used to estimate Vitamin E in the milk.

### Statistical analysis

Mean and standard error were calculated and tested for significance using t-test (Snedecor and Cochran) [15].

## Results

### Antioxidative profiling

**Figure-1:**
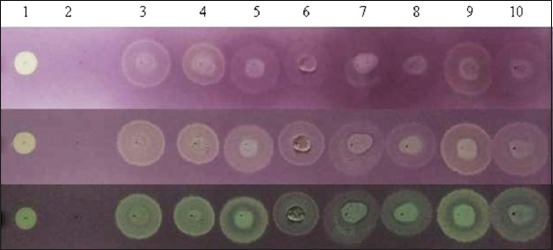
Antioxidant activity as elucidated by qualitative 2, 2-Diphenyl-1-picrylhydrazyl (DPPH) test using F_254_ thin-layer chromatography silica coated-aluminum plates. (a) Color developed immediately after DPPH spray; (b) yellow spot appearing after 30 min., (c) yellow spot after 18 h. Lane 1 – ascorbic acid (+ve control); lane 2 – methanol (−ve control); lanes 3 and 4 – skim milk of indigenous hill cattle; lanes 5 and 6 – skim milk of Jersey crossbred cattle; lanes 7 and 8 – skim milk of Gaddi goat; lanes 9 and 10 – skim milk of non-Gaddi goats. Each spot was developed from 10 µl of samples.

This study involves analysis of the composition of raw skimmed milk of indigenous cattle and goats of NWHR. Total phenol and antioxidant activities in cattle and goat milk are presented in Table-1. DPPH radical scavenging activity was significantly (p<0.05) higher in indigenous hill cattle milk. The emergence of yellow spots revealed antioxidant activity in skim milk (Figure-1). Total phenol content was significantly (p<0.05) higher in indigenous hill cattle, followed by non *-Gaddi* goat, Jersey crossbred cattle, and *Gaddi* goat milk.

**Table 1:** Total phenol, DPPH antioxidant activities, and protein in cattle and goat milk.

Animal type	Total phenol (mg TAE/100 ml)	DPPH (%)	Protein (%)
Indigenous hill cattle (n=6)	60.40^A^±0.61	16.34^A^±0.31	1.33^A^±0.01
Jersey crossbred cattle (n=6)	41.00^B^±0.51	9.06^B^±0.36	1.03^B^±0.02
Non-*Gaddi* goat (n=6)	43.43^B^±0.31	6.48^C^±0.25	0.76^C^±0.05
*Gaddi* Goat (n=6)	38.93^C^±0.40	6.93^C^±0.46	0.81^C^±0.01

n=Number of samples analyzed; values with different superscripts within the column are statistically different (p<0.05). DPPH=2, 2-Diphenyl-1-picrylhydrazyl

### Protein profiling

**Figure-2:**
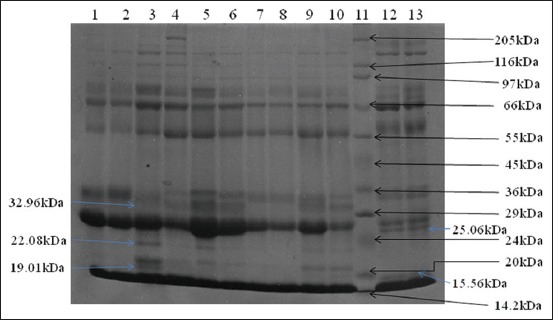
Sulfate-polyacrylamide gel electrophoresis analysis of skimmed milk proteins: Lanes 1, 2, 7, 8: Local non-*Gaddi* goats; lanes 3, 4, 5, 6, 9, 10: *Gaddi* goats; lane 12: Local hill cattle; lane 13: Jersey crossbred cattle; lane 11: Molecular protein marker.

Average protein concentration in raw skimmed milk was 1.33±0.01, 1.03±0.02, 0.76±0.05, and 0.81±0.01%, respectively, in indigenous hill cattle, Jersey crossbred cattle, non *-Gaddi* goat, and *Gaddi* goat (Table-1). Protein in skim milk was significantly (p<0.05) higher in indigenous hill cattle. The protein concentration in Jersey crossbred cattle skimmed milk was significantly (p<0.05) low as compared to skimmed milk proteins in indigenous hill cattle. No significant differences were observed in skim milk protein contents of non *-Gaddi* with reference to *Gaddi* goats. SDS-PAGE profile of skim milk proteins is shown in Figure-2.

SDS-PAGE protein profiles did not show any difference among indigenous hill cattle and Jersey crossbred animals. A total of 12 protein bands of varying size and intensity were noted in skimmed milk of *Gaddi* goat. SDS-PAGE revealed the presence of 19.01, 22.08, and 32.96 kDa protein in *Gaddi* goats skimmed milk, but not in non *-Gaddi* goat milk. Moreover, these proteins were also missing in cattle skim milk. Two proteins of 15.56 and 25.06 kDa were present in local and crossbred cattle skimmed milk, but not in goat skimmed milk (Figure-2).

### Vitamin profiling

A variation was noted in vitamin content in the milk of different species (Table-2). Vitamin A content was lower in non *-Gaddi* goat, but comparable in other species. Vitamin C content was lower in *Gaddi* goat, but higher in Jersey crossbred cattle milk. Vitamin E content was highest in non *-Gaddi* goats and lowest in *Gaddi* goat milk.

**Table 2:** Vitamin content in cattle and goat milk.

Animal type	Vitamin C (mg/100 ml)	Vitamin A (μ/ml)	Vitamin E (μ/ml)
Indigenous hill cattle (n=80)	1.31^A^±0.05	1.00^A^±0.01	1.53^C^±0.05
Jersey crossbred cattle (n=80)	1.45^A^±0.10	1.04^A^±0.02	1.68^B^±0.07
Non-*Gaddi* goat (n=80)	1.38^A^±0.06	0.95^B^±0.04	1.83^A^±0.11
*Gaddi* Goat (n=80)	0.88^B^±0.06	1.03^A^±0.02	1.14^D^±0.06

n=Number of samples analyzed; values with different superscripts within the column are statistically different (p<0.05)

## Discussion

Milk is an important component of human nutrition and is a source of probiotics, postbiotics, and high biological value proteins [16], which during processing or digestion in gastrointestinal tract is disassembled into peptides. The peptides confer diverse biological activities including antimicrobial, angiotensin-transforming enzymes inhibition, scavenging of free radicals, opioid, and immunomodulation [17-19].

There is an increasing interest in using phenolics due to their therapeutic properties, antioxidant, and free radical scavenging potential [20,21]. In addition, ingested phenolic compounds prevent oxidation of polyunsaturated fatty acids [22]. Notably, the dietary phytonutrients and their gut microbial metabolites affect composition of milk and general health of the host [23,24]. O’Conell and Fox [25] noted that phenolic profile of milk greatly changes depending on the type and composition of nutrients consumed by the animals. A total of 127 phenolic compounds were detected in the milk of cows that grazed on different plants, whereas 87 phenolic metabolites were present in cows fed forage hay [26].

The DPPH free radical is highly stable and reacts with substances that can donate H^+^ ions. DPPH is used to determine antioxidative properties of biomolecules. In this study, DPPH was dissolved in methanol as proteins in aqueous media may not easily diffuse to target DPPH radicals. To eliminate the effect of methanol, TLC-based dot assay of DPPH scavenging was used. The appearance of yellow spot indicates reduction of DPPH radical. Compared with control, skimmed milk showed DPPH scavenging activities after 30 min, and more intense spot thereafter representing efficient scavenging activity (Figure-1). The TLC assay showed an imprecise intensity in the first 30 min after DPPH spray. This indicated that the milk has both slow- and fast-reacting antioxidants as has already been noted in the earlier study [27].

SDS-PAGE is an important and simpler tool widely used to identify and elucidate mutations, or polymorphism in DNA and RNA or proteins among different samples or species [28]. In the present study, cattle and goat skimmed milk proteins were analyzed by SDS-PAGE. Protein profiles did not reveal any difference among indigenous hill cattle and Jersey crossbred animals. However, comparative studies indicated a marked difference in protein profiles of goats and cow skimmed milk. SDS-PAGE revealed the presence of 19.01, 22.08, and 32.96 kDa proteins in *Gaddi* goats skimmed milk, but not in non *-Gaddi* goat milk. Moreover, these proteins were also undetectable in cattle skim milk. Use of goat milk in infant’s diet, as a source of proteins, has significantly increased in recent past as an alternative to cow milk as A-1 type cattle milk contains proteins implicated in hypersensitivity reactions, cardiovascular, and metabolic diseases such as Type 2 diabetes [29].

There is surge in the use of animal milk other than cattle as an alternative dietary supplement because of certain adverse health effects associated with A-1 milk of exogenous or crossbred cows [30]. Goat milk is thought to have a low allergenic potential compared to cow milk based on characteristics of αS1-casein [31]. It is revealed that daily use of goats’ milk in the diet of immuno-compromised aged patients acts as a downregulator of acute inflammation, reduces the embellished basal secretion of interleukin (IL)-8 and IL-6 acute response, and exerts a moderate downregulation of IL-1β and tumor necrosis factor-α production [32].

Goat milk has an anti-aging effect, hence used in dry skin therapeutic formulations. Goat milk is documented to exhibit biological process of oxidation-reduction, cellular components of extracellular exosomes, and molecular function of poly (A) RNA binding [33]. Further, the study reveals that cattle and goat whey proteins are involved in disease, metabolism, and immune pathways with different number and types [33]. Although further studies are warranted, we envisage that bioactive metabolites present in milk of *Gaddi* goats might offer antiaging and pro-health effects. The present work provides useful insights into composition of hill cattle and native goat milk proteins with beneficial health benefits.

## Conclusion

Milk is used for centuries due to its nutritional, therapeutic, and anti-aging properties. The present study will be useful to differentiate bioactive metabolites in goat and cow milk. Presence of specific proteins in some goat’s milk is an interesting observation and needs further research to decipher biological significance of these proteins. In addition, studies should also be carried out to discover the use of biologically active milk components as nutraceuticals or dietary supplements to boost well-being and prevent aging and associated health problems.

## Authors’ Contributions

VS, JBD, and GM performed laboratory analysis work. BS, RS, and NS contributed to milk samples collection, experiment designs, and statistical analysis. All authors read and approved the final manuscript.
